# Distinctive pathological and clinical features of lung carcinoids with high proliferation index

**DOI:** 10.1007/s00428-017-2177-0

**Published:** 2017-06-19

**Authors:** Caterina Marchiò, Gaia Gatti, Federica Massa, Luca Bertero, Pierluigi Filosso, Giuseppe Pelosi, Paola Cassoni, Marco Volante, Mauro Papotti

**Affiliations:** 1 Department of Medical Sciences, University of Turin, Via Giuseppe Verdi, 8, 10124 Turin, Italy; 2 Pathology Division, AOU Città della Salute e della Scienza di Torino, Via Santena 7, 10126 Turin, Italy; 3 Pathology Division, San Luigi Hospital, Regione Gonzole 10, 10043 Orbassano, Italy; 4 Department of Surgical Sciences, University of Turin, Via Giuseppe Verdi, 8, 10124 Turin, Italy; 5 Department of Oncology and Hemato-Oncology, Università degli Studi di Milano, Via Festa del Perdono, 7, I-20122 Milan, Italy; 6 Inter-hospital Pathology Division, Science & Technology Park, IRCCS MultiMedica Group, Milan, Italy; 7 Department of Oncology, University of Turin, Via Giuseppe Verdi, 8, 10124 Turin, Italy

**Keywords:** Carcinoid, Lung, WHO classification, Ki-67, Heterogeneity, Prognosis

## Abstract

Typical (TCs) and atypical carcinoids (ACs) are defined based on morphological criteria, and no grading system is currently accepted to further stratify these entities. The 2015 WHO classification restricts the Ki-67 role to biopsy or cytology samples, rather than for prognostic prediction. We aimed to investigate whether values and patterns of Ki-67 alone would allow for a clinically meaningful stratification of lung carcinoids, regardless of histological typing. Ki-67 proliferation index and pattern (homogeneous versus heterogeneous expression) were assessed in a cohort of 171 TCs and 68 ACs. Cases were subdivided into three Ki-67 ranges (<4/4–9/≥10%). Correlations with clinicopathological data, univariate and multivariate survival analyses were performed. The majority of cases (61.5%) belonged to the <4% Ki-67 range; 25.1 and 13.4% had a proliferation index of 4–9% and ≥10%, respectively. The <4% Ki-67 subgroup was significantly enriched for TCs (83%, *p* < 0.0001); ACs were more frequent in the subgroup showing Ki-67 ≥ 10% (75%, *p* < 0.0001). A heterogeneous Ki-67 pattern was preferentially seen in carcinoids with a Ki-67 ≥10% (38%, *p* < 0.02). Mean Ki-67 values ≥4 and ≥10% identified categories of poor prognosis both in terms of disease-free and overall survival (*p* = 0.003 and <0.0001). At multivariate analysis, the two thresholds did not retain statistical significance; however, a Ki-67 ≥ 10% identified a subgroup of dismal prognosis even within ACs (*p* = 0.03) at univariate analysis. Here, we describe a subgroup of lung carcinoids showing brisk proliferation activity within the necrosis and/or mitotic count-based categories. These patients were associated with specific clinicopathological characteristics, to some extent regardless of histological subtyping.

## Introduction

Carcinoid tumours of the lung are rare primary lesions accounting for 0.2% (atypical carcinoids, AC) to 2% (typical carcinoids, TC) of resected lung cancers. According to the 2015 WHO classification [1], they make up the morphologically defined group of neuroendocrine tumour (NETs) based upon cytological traits, mitotic count and necrosis. Lung NETs comprise low- to intermediate-grade forms (TCs and ACs, respectively), clearly segregated from high-grade malignancies, namely large cell NE carcinomas and small cell carcinomas. Carcinoid tumours, however, translate into a broad clinicopathologic spectrum characterized by highly variable outcomes, which are currently best stratified according to the 2015 WHO classification and 8th edition TNM staging [1]. Surgery is the best treatment modality choice for both TCs and ACs [2], with TCs usually exhibiting long life expectancy with 5- and 10-year survival rate by far over 90% [3]. Conversely, ACs exhibit a more aggressive clinical course and a 5-year survival rate ranging from 56 to 87%, which is lower in node positive patients, suggesting that this subgroup could benefit from some adjuvant treatment [4–8].

In pulmonary NETs, whether biopsy samples or surgical specimens, no grading system is currently accepted for further stratifying single tumour entities beyond the pure tumour histological typing. Indeed, the grading system proposed for gastroenteropancreatic (GEP) NETs does not apply to thoracic NETs, nor Ki-67 plays a role in tumour classification. In the 2015 WHO classification, the Ki-67 prognostic role is not established and is considered valuable in biopsy or cytology samples only [1]. As a matter of fact, several groups have confirmed the significant prognostic value of Ki-67 in lung NETs [9], with a 4–5% cut-off being the most widely employed for the sake of reproducibility and reliability [10] compared to mitotic count and necrosis, especially when dealing with biopsy specimens [11, 12].

However, in multivariate survival analysis, Ki-67 seems to add little value to histological typing [13, 14] when a 4–5% cut-off is adopted, but results from several studies are still inconclusive. In order to integrate rather than to replace the histological classification with the information stemming from Ki-67 index determination, Rindi and co-workers have proposed an accurate and innovative grading system for pulmonary NETs (G1 to G3) where mitotic count, necrosis and Ki-67 index were jointly combined on the basis of lung-specific cut-off values [15]. At the histological level, only TCs by morphology turned out G1, while ACs and the LCNEC/SCLC group crossed G1 to G3 and G2 to G3 categories, respectively, confirming the inherent heterogeneity in behaviour of intermediate- to high-grade lung NETs [15].

In the GEP area, NET-G3 retaining a well-differentiated morphology but showing proliferation rates consistent with NE carcinomas, either in the whole tumour or in focal areas only, have recently been recognized [16, 17]. These tumours are associated to genetic alterations of well-differentiated tumours, but with a clinical behaviour intermediate between G2 NETs and more conventional NE carcinomas [18–20]. This phenomenon would identify secondary high-grade NE tumours evolving from pre-existing lower grade carcinoids, as recently demonstrated in the thymus [21]. In the lung, LCNECs with carcinoid-like appearance have been identified [22, 23], but little is known about the clinical impact of carcinoid tumours, as defined by current diagnostic guidelines, showing high Ki-67 indices.

Based on these premises, in this study, we sought to investigate the prevalence of carcinoid tumours showing brisk proliferation activity and to define whether values and patterns of Ki-67 labelling alone would allow for a clinically meaningful stratification of a large cohort of lung carcinoids, regardless of the histological type. This could be propaedeutic to the identification of low- to intermediate-grade NETs in the lung that may have the potential to evolve to a higher grade disease, thus sustaining the paradigm shift to secondary high-grade NETs even in the lung.

## Materials and methods

### Cohort and Ki-67 scoring

The pathology files of the AOU Città della Salute e della Scienza di Torino and San Luigi Hospital were interrogated for a diagnosis of carcinoid tumour on surgical specimen, and a total of 239 consecutive specimens were retrieved. All original H&E slides were reviewed by four of the authors (CM, GG, MV, MP) to confirm the diagnostic categorization, including re-evaluation of the mitotic count and Ki-67 index, the latter on newly stained slides (if unavailable or suboptimal). The cohort comprised 171 TCs and 68 ACs, defined according to the WHO 2015 criteria [1]. The study has been approved by the local ethical committee (Department of Oncology at San Luigi Hospital, number 17975, October 14th 2015). Discordant cases were discussed at a multi-headed microscope to reach agreement. We manually scored Ki-67 labelling index by counting at least 1000 cells. We also recorded whether the pattern of Ki-67 labelling was homogeneous throughout tumour samples or whether hotspot areas could be clearly identified (heterogeneous Ki-67 pattern). Whenever hotspot areas were detected, the highest recorded value was taken into account to perform statistical correlative analyses. Hotspot areas were defined as *foci* of tumour cells selected at low magnification (40×) based on an overcrowding of positive nuclei that led to a much higher count than the mean Ki-67 index evaluated on the overall tumour cell population. No specific cut-offs were adopted to identify hotspots. This definition allowed to identify areas of heterogeneous proliferation even within the context of TCs, where a proliferation count as low as 1 to 4% is usually encountered.

A dedicated database was constructed where clinicopathological features were annotated, including follow-up data.

### Statistical analysis

Cases were subdivided according to three different Ki-67 ranges (<4, 4–9, ≥10%) and correlation with histopathological features; pattern of Ki-67 and follow-up data were performed by Chi square test. The 4% threshold for Ki-67 is one of the most frequently adopted in literature [14, 15, 24–26], while the ≥10% cut-off was chosen based on the mean Ki-67 value of 9% observed in this series of AC (otherwise belonging to the 10–20% range mentioned for carcinoids in the current WHO classification [1]). A *p* < 0.05 value was considered statistically significant. Univariate time to progression and overall survival analyses were performed with the Kaplan-Meier method, and the log-rank test was employed to compare survival curves. Cox proportional hazards regression model was also used in multivariate survival analysis.

## Results

### Clinicopathological features

**Fig. 1:**
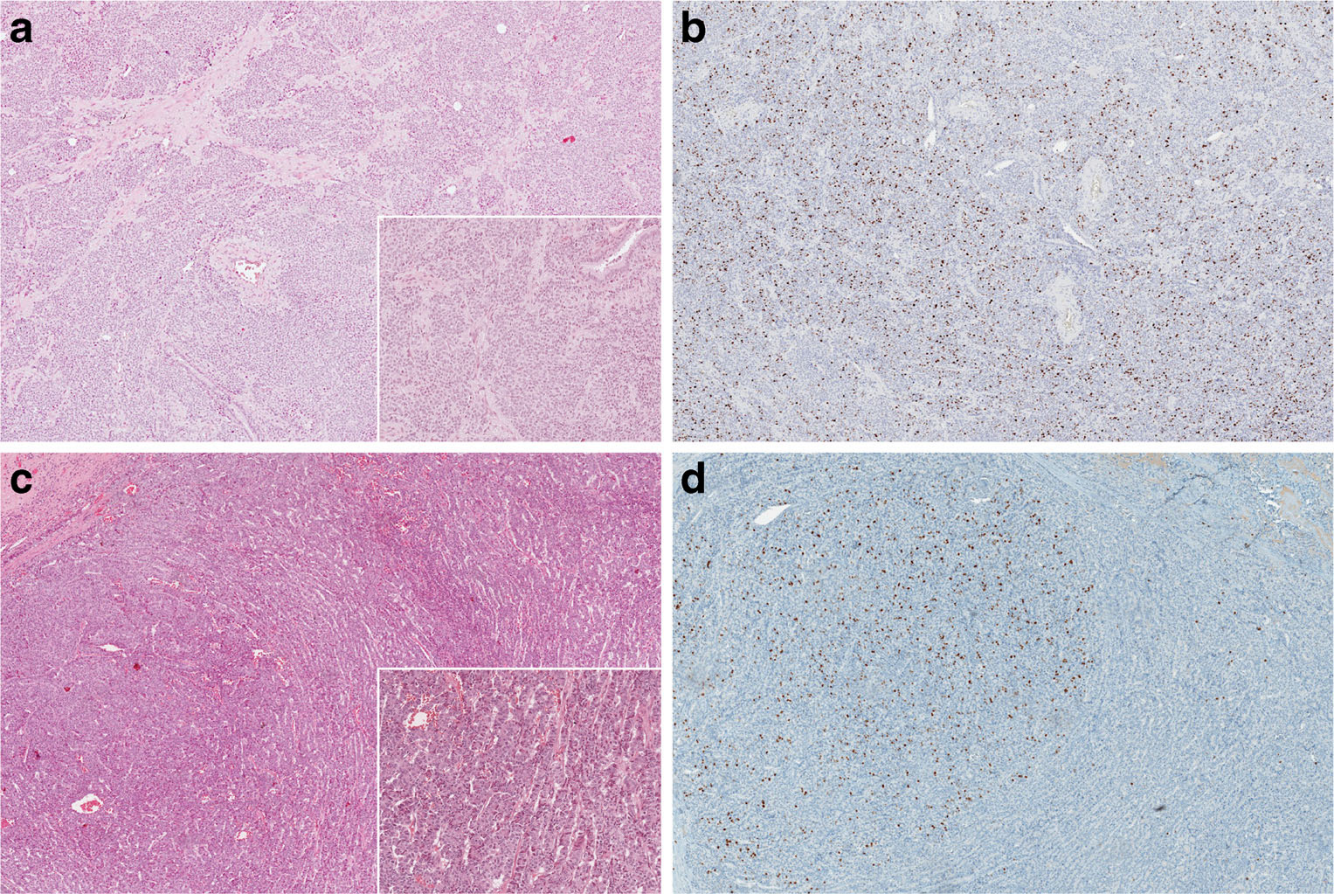
Atypical carcinoids showing different Ki-67 patterns (homogenous versus heterogeneous). ACs with organoid and trabecular growth patterns (**a** and **c**: H&E 40×, inset 100×) showing a homogenously (**b**: 40×) and heterogeneously (**d**: 40×) increased Ki-67 index, in both cases up to 12%

TCs and ACs showed a mean Ki-67 value of 2.8 and 9%, respectively, ranging from 1 to 18% for TCs and 1 to 43% for ACs (Fig. 1). Of note, four cases of ACs (1.7% of the total, 5.9% of the ACs) displayed a mean proliferation index of ≥25%.

Stratification of the tumour cohort according to 4 and 10% Ki-67 cut-off thresholds is shown in Table 1. The large majority of cases (147/239, 61.5%) pertained to the lowest Ki-67 range (<4%), 60 cases (25.1%) had a proliferation index between 4 and 9%, and a minor subgroup (32/239, 13.4%) displayed a Ki-67 labelling index of ≥10%.

**Table 1:** Clinicopathological features stratified according to different classes of proliferation, as defined by Ki-67 cut-offs of 4 and 10%

Parameter		Ki-67 < 4% (#147)	Ki-67 4–9% (#60)	Ki-67 ≥ 10% (#32)	*p* value
Sex	M	66	18	16	0.08
	F	81	42	16	
Age	Median	56	55	64	0.45
Histological type	TC	122	41	8	<0.0001
	AC	25	19	24	
pT	pT1–2	133	56	26	0.35
	pT3–4	14	4	5	
pN	pN0	113	49	21	0.21
	pN+	28	10	10	
Ki-67 pattern	Homogeneous	80	32	18	0.02
	Heterogeneous	13	8	11	
Vascular invasion	Present	34	17	17	0.001
	Absent	101	33	11	
Pleura	PL0	100	28	20	0.001
	PL+	3	0	5	
Patient status	NED/DOC	127	51	20	<0.0001
	AWD/DOD	11	5	10	
Survival data	Median TTP (months)	Undefined	Undefined	101	0.0052
	Median OS (months)	244	Undefined	122	<0.0001

*M* male, *F* female, *TC* typical carcinoid, *AC* atypical carcinoid, *NED* no evidence of disease, *DOC* died of other unrelated causes, *AWD* alive with disease, *DOD* died of disease, *OS* overall survival, *TTP* time to progression

**Fig. 2:**
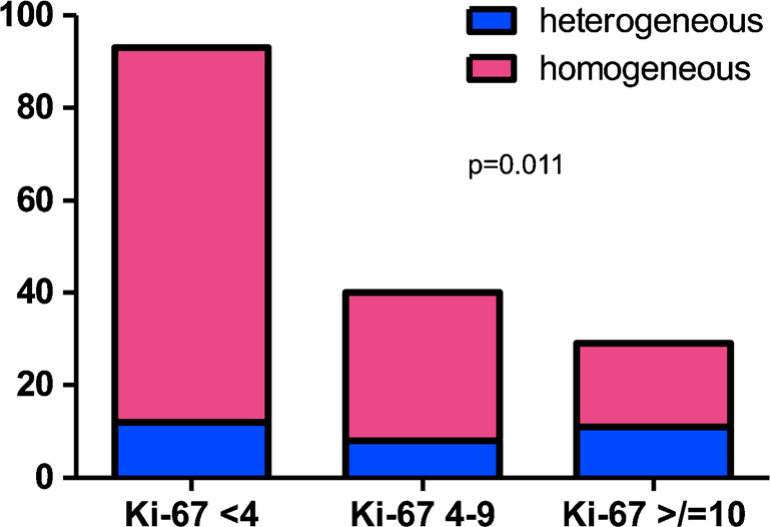
Relative prevalence of Ki-67 pattern (homogeneous versus heterogeneous distribution) in lung carcinoids. A homogeneous Ki-67 labelling pattern was significantly more represented in the low (<4%) Ki-67 subgroup (86%, *p* < 0.02, Chi square test) and in the intermediate (4–9%) Ki-67 subgroup (80%, *p* < 0.02, Chi square test), whereas a heterogeneous Ki-67 pattern was preferentially seen in the subgroup of carcinoids with a proliferation index of ≥10% (38%, *p* < 0.02, Chi square test)

The low Ki-67 subgroup (<4%) was significantly enriched for TCs (122/147, 83%, *p* < 0.0001, Table 1), whereas ACs were more frequently found in the subgroup showing a mean Ki-67 of ≥10% (24/32, 75%, *p* < 0.0001, Table 1). Nevertheless, eight cases with a Ki-67 higher than 10% were classified as TC, according to the morphological criteria. A homogeneous Ki-67 labelling pattern was a significant feature of the low (<4%) Ki-67 subgroup (80/93, 86%, *p* < 0.02, Table 1, Fig. 2) as well as of the intermediate (4–9%) Ki-67 subgroup (32/40, 80%, *p* < 0.02, Table 1), whereas a heterogeneous Ki-67 pattern was preferentially seen in the subgroup of carcinoids with a proliferation index of ≥10% (11/29 assessable cases, 38%, *p* < 0.02, Table 1, Figs. 1 and 2).

Vascular invasion and pleural involvement were relatively more represented in the carcinoid subgroup with Ki-67 ≥ 10%, and the differences among subgroups were statistically significant (*p* = 0.001, Table 1).

### Patient outcome

#### Univariate analyses

**Fig. 3:**
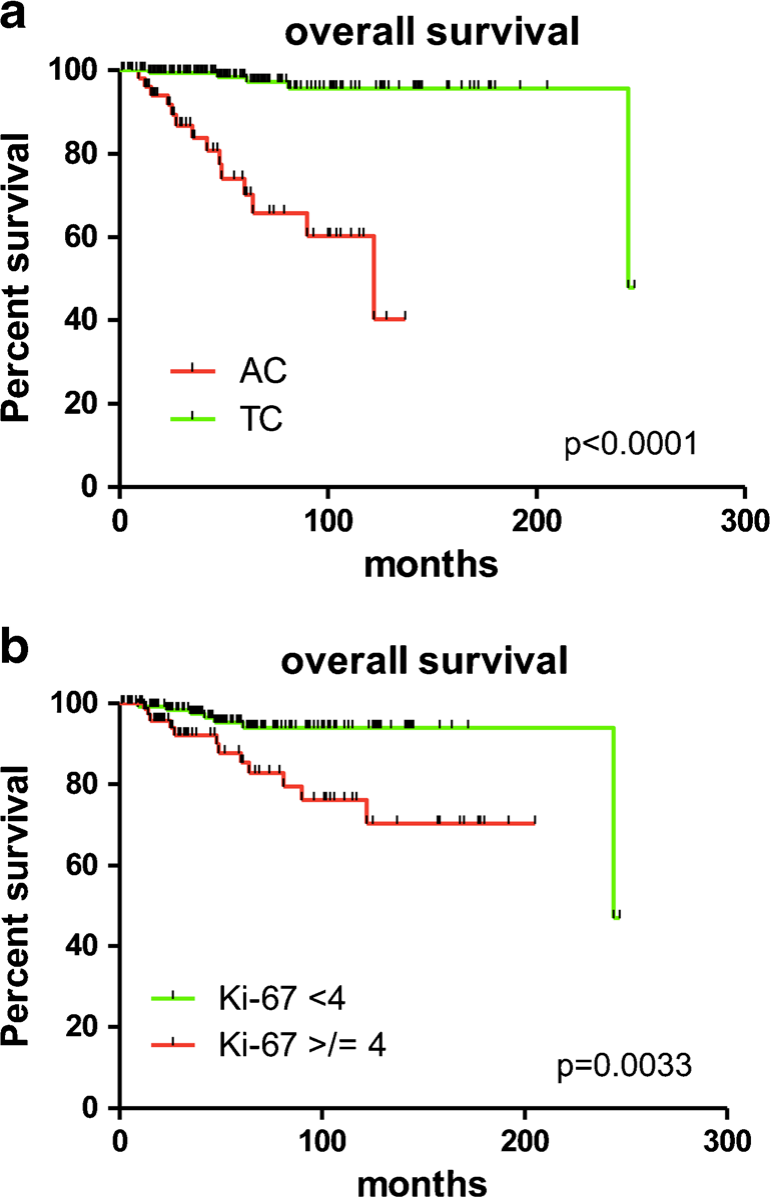
Kaplan-Meier survival curves in lung carcinoids segregated according to histological type (**a**) and Ki-67 > 4% (**b**). **a** Patients with a TC diagnosis had an excellent overall survival, which was significantly better compared to patients affected by ACs (*p* < 0.0001). **b** The 4% cut-off on the whole tumour cohort effectively split patients into worse categories for tumours displaying Ki-67 higher than 4% (*p* = 0.003)

Patients with a TC diagnosis had an excellent disease-free and overall survival, which was significantly better compared to patients affected by ACs (*p* < 0.0001, Tables 2 and 3, Fig. 3).

**Table 2:** Univariate and multivariate time to progression survival analysis

Parameter	Carcinoids—time to progression survival (#219)				ACs—time to progression survival (#58)	
	Univariate		Multivariate		Univariate	
	HR (CI)	*p*	HR (CI)	*p*	HR (CI)	*p*
Sex (M vs F)	4.015 (1.697–9.785)	0.002	0.12 (0.03–0.44)	0.001	3.424 (1.243–9.430)	0.017
Age (above vs below median)	1.570 (0.664–3.712)	0.3	–	–	0.450 (0.155–1.310)	0.14
Histological type (AC vs TC)	55.87 (18.13–172.2)	<0.0001	22.90 (4.628–113.34)	0.0001	–	–
Rindi’s grade (2 vs 1)	50.01 (11.53–217.0)	<0.0001	0.261 (0.066–1035)	0.22	1.193 (0.445–3.199)	0.72
Size (above vs below mean)	1.438 (0.570–3.628)	0.59	–	–	1.170 (0.406–3.366)	0.7
Clinical stage (all others vs IA/B)	2.001 (0.792–5050)	0.14	–	–	1.703 (0.615–4.716)	0.3
Nodal status (N+ vs N0)	7093 (2327–21.61)	0.0006	3.374 (0.684–13.63)	0.036	1.921 (0.764–5.232)	0.2
Vascular invasion (VI+ vs VI−)	4.709 (1.684–13.16)	0.003	–	–	1.634 (0.559–4.776)	0.36
KI-67 (≥4 vs <4)	3.994 (1.58–10.0)	0.003	3.374 (0.684–16.63)	0.13	1.252 (0.450–3.475)	0.66
Ki-67 (≥10%)	17.121 (4.32–68.56)	<0.0001	0.767 (0.198–2.95)	0.7	1.772 (0.610–5.108)	0.28
Necrosis (present vs absent)	369 (65.42–2082)	<0.0001	2.186 (0.622–7.678)	0.2	2.656 (0.925–7.625)	0.07

**Table 3:** Univariate and multivariate overall survival analysis

Parameter	Carcinoids—overall survival (#219)				ACs—overall survival (#58)	
	Univariate		Multivariate		Univariate	
	HR (CI)	*p*	HR (CI)	*p*	HR (CI)	*p*
Sex (M vs F)	3.429 (1.345–8.740)	0.01	0.526 (0.218–1.271)	0.15	2.477 (0.853–7.187)	0.09
Age (above vs below median)	1.617 (0.665–4.08)	0.28	–	–	1.336 (0.435–4.097)	0.6
Histoloigc type (AC vs TC)	39.63 (12.37–127.9)	<0.0001	3.029 (0.923–9.939)	0.06	–	–
Rindi’s grade (2 vs 1)	40.14 (8.760–183.9)	<0.0001	0.383 (0.111–1.441)	0.15	1.325 (0.461–3.828)	0.6
Size (above vs below mean)	7.437 (0.539–3.823)	0.52	–	–	1.22 (0.389–3.828)	0.7
Clinical stage (all others vs IA/B)	1.324 (0.486–3.605)	0.3	–	–	1.401 (0.450–4.355)	0.6
Nodal status (N+ vs N0)	5.109 (1.503–16.70)	0.007	2.930 (1.161–7.546)	0.02	2.206 (0.733–6.642)	0.16
Vascular invasion (VI+ vs VI−)	7.646 (2.531–23.10)	<0.0001	–	–	4.128 (1.251–13.63)	0.02
KI-67 (≥4 vs <4)	4.31 (1.624–11.45)	0.003	2.879 (0.837–9.904)	0.09	1.451 (0.49–4.29)	0.5
Ki-67 (≥10%)	36.79 (8.868–152)	<0.0001	1.949 (0.629–6.036)	0.24	3.53 (1.128–11.08)	0.03
Necrosis (present vs absent)	281 (46.50–1698)	<0.0001	2.004 (0.627–6.411)	0.24	3.094 (0.977–9.793)	0.05

**Fig. 4:**
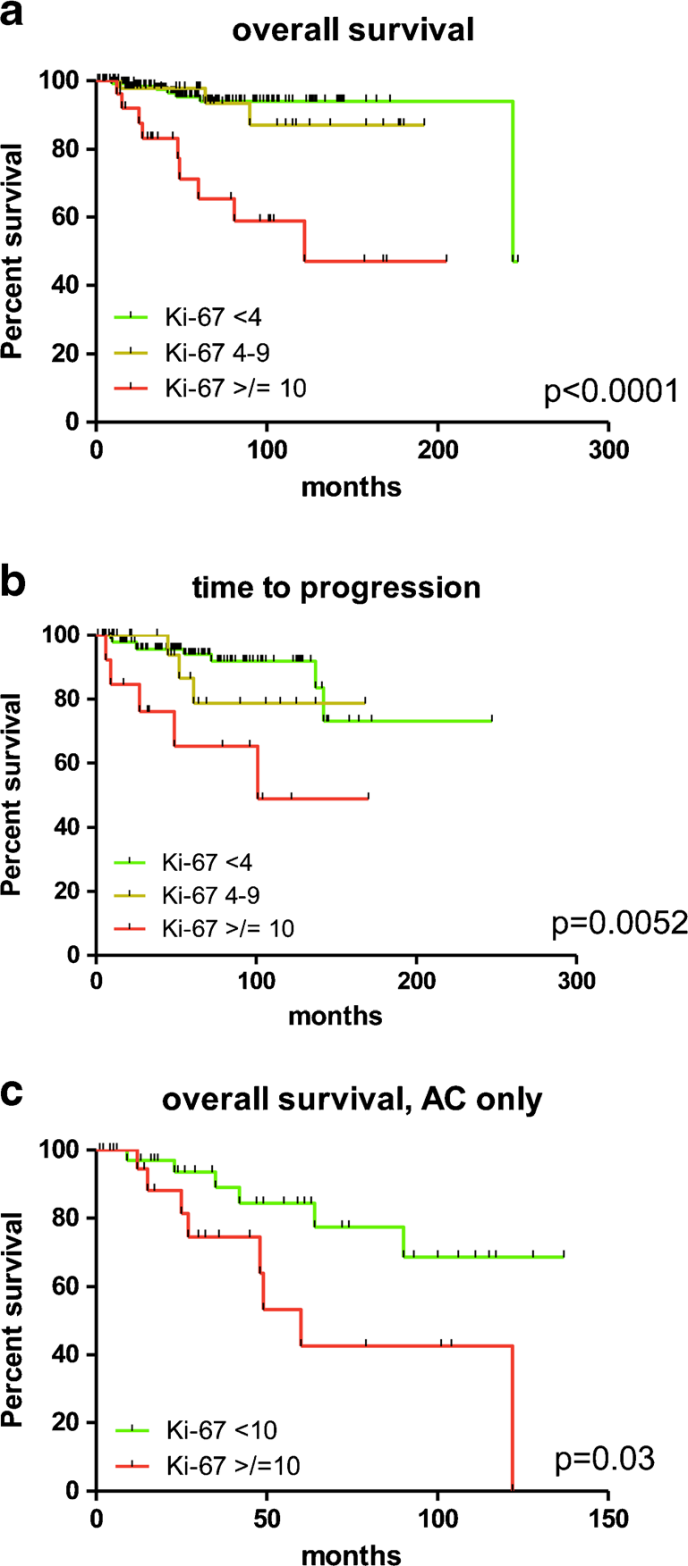
Kaplan-Meier survival curves in lung carcinoids segregated according to distinct Ki-67 thresholds (4 and 10%). Analysis of patients’ outcome in terms of overall survival (**a**) and time to progression (**b**) in the whole cohort of lung carcinoids stratified according to 4 and 10% cut-offs. Of note, the difference in terms of outcome between the categories of <4 and 4–9% was not statistically significant for either overall survival or time to progression (*p* = 0.40 and *p* = 0.32, respectively). **c** Subgroup analysis restricted to atypical carcinoids segregated according to the 10% cut-off, which significantly predicted the ultimate outcome of patients (*p* = 0.03) even in the sole AC histological type

When investigating Ki-67, we first dichotomized cases according to a 4% cut-off on the whole tumour cohort finding that it effectively split patients into worse categories in terms of both disease-free and overall survival for tumours displaying Ki-67 higher than 4% (*p* = 0.003, Tables 2 and 3; Fig. 3). The same held true when stratifying cases by using the 10% cut-off threshold, with two different prognostic populations for both time to progression and overall survival (*p* < 0.0001, Tables 2 and 3). In addition, when the whole tumour cohort was stratified into three groups according to the 4 and 10% cut-off thresholds, we observed that outcome of patients with tumours pertaining to the three relevant Ki-67 categories (<4, 4–9, ≥10%) was different in terms of both overall survival and time to progression (*p* = 0.0052 and <0.0001, respectively; Table 1 and Fig. 4), with cases displaying a Ki-67 ≥ 10% running a poorer survival. Of note, the difference in terms of outcome between the categories of <4% and 4–9% was not significantly different for either overall survival or time to progression (*p* = 0.40 and *p* = 0.32, respectively). Importantly, the ≥10% cut-off was capable of significantly predicting patients’ outcome in terms of overall survival (*p* = 0.03, Fig. 4) even in the sole AC subgroup (accounting for the largest number of tumours with a relatively higher Ki-67).

Finally, male sex, G2 Rindi’s grade, presence of vascular invasion or necrosis, and positive nodal status were significantly associated with adverse prognosis at both time to progression and overall survival.

#### Multivariate analysis

Male sex, nodal status and histological typing were independent prognostic factors at multivariate analysis, whereas the two Ki-67 cut-off thresholds of ≥4 and ≥10% did not retain statistical significance upon multivariate analysis (Tables 2 and 3). The subgroup of ACs was not powered enough to perform a multivariate analysis.

## Discussion

In this study, we showed that up to 13% of lung carcinoids display a Ki-67 proliferation index of ≥10% and that 6% of ACs may show Ki-67 values around 25%. Most importantly, a 10% cut-off for Ki-67 labelling index provided a clinically meaningful stratification of carcinoid tumours of the lung even within the subgroup of ACs, thereby providing evidence that proliferation index assessed by Ki-67 would add useful prognostic information to histological subtyping.

It is currently a matter of debate whether the 2015 WHO classification [1] is adequate enough to encompass the spectrum of lung NETs. This classification has substantially confirmed the four categories already crystallized in the two previous editions: it is solely based on histological parameters [27], and the threshold of 10 mitoses/2 mm^2^ is the criterion used to take pulmonary carcinoids apart from NE carcinomas [1]. Here, we show that the rare group of carcinoid tumours is biologically heterogeneous, including a subgroup of lesions with a relatively high proliferative potential as highlighted by the Ki-67 index. From a classification standpoint, the even rarer AC category is monolithic (necrosis and/or 2 to 10 mitoses/2mm^2^), even if a cut-off of six mitoses has been proposed, but not envisaged, to separate tumours with different life expectation [28]. The only diagnostic alternative is the category of high-grade neuroendocrine carcinomas, either LCNECs or SCLCs, which is however ruled out upon mitotic count. Interestingly, even LCNECs may show morphologic [22, 23] and molecular [23] features rather akin to ACs, supporting the view of a grey-zone in lung NETs where a biological continuum could probably be more effective than categorical criteria for classification purposes. Most importantly, the optimal treatment options for ACs after surgery, particularly in the metastatic setting, are highly debated and variably including close follow-up, systemic therapies different from or comprising platinum or even biological drugs (somatostatin analogues or m-TOR inhibitors).

Based on this evidence, we reasoned that rather than forcing the four-tier histological classification, an approach based on proliferation activity could be beneficial to best depict the clinical variety within carcinoid tumours, thereby integrating a purely histological classification. At present, over 2000 lung NET cases have been published having Ki-67 investigated as part of the morphological and phenotypical description [9], and most of these studies recognize the usefulness of Ki-67 index reporting. A multicentre study on almost 400 cases proposed a grading system for lung NETs incorporating the Ki-67 index alongside mitotic count and necrosis evaluation [15].

In our study, we wanted to ascertain whether Ki-67 alone, not engaged in a grading system, would allow a prognostic classification within the category of carcinoid tumours with the specific aim of identifying a niche of carcinoid tumours (defined according to the official parameters) having a proliferation index above the expected values. Our results clearly show a prognostic separation in terms of both overall survival and time to progression when cut-offs of 4 and 10% are adopted. Our analysis also revealed that the tumours with either <4 or 4–9% proliferation indices showed comparable survival rates, thus suggesting the 10% as a more suitable cut-off to identify lung carcinoids with a clinically meaningful brisk proliferation activity.

The adoption of Rindi’s grade (grade 2 versus 1) showed a significant correlation with outcome in univariate but not in multivariate analysis. Although also the Ki-67 thresholds here analysed did not reach statistical significance in multivariate analysis, it is interesting to note that the 10% cut-off was able to tease out a category of tumours harbouring dismal prognosis even within the restricted category of ACs, whereas Rindi’s grade did not hold significant prognostic information in this subset of patients.

Importantly, a heterogeneous Ki-67 proliferation pattern was significantly more common in the category of cases displaying a Ki-67 labelling index ≥10%. This is in line with recent reports suggesting that intra-tumour heterogeneity holds per se a negative prognostic impact, as shown across several distinct diseases [29].

We documented in our study that a non-negligible number of life-threatening ACs showed quite unexpected high Ki-67 indices, which overlapped with those reported for LCNECs [1, 9], even if not fulfilling the relevant mitotic count for this tumour category. Although Ki-67 levels and mitoses do not entirely reflect the same cell cycle-associated event, it is known that pancreatic NETs with discrepant Ki-67 and mitotic indices bear different impacts on tumour grading [30]. Moreover, we speculate that these ACs with high Ki-67 index could harbour a potential of transition towards LCNECs with similar molecular profiles, either synchronously or metachronously, as recently demonstrated in thymus ACs [21]. Furthermore, a recent study by Simbolo et al. [31] has also shown that rare lung LCNECs resembling carcinoids harbour *MEN1* mutations—the most frequent alteration in ACs—along with *TERT*, *SDHA*, *RICTOR*, *PIK3CA*, *MYCL* and *SRC* gains are shared with high-grade carcinomas. This is in line with a paradigm shift, even in the lung, that envisages the development of secondary high-grade NETs from pre-existing lower grade NETs, as demonstrated in the thymus [21]. Regrettably, the numerosity of outlier tumour carcinoids, i.e. either highly proliferating or harbouring genetic features of aggressive disease (*TP53* mutations, hypermutated profile), was too limited in the recent study reported by Simbolo et al. [31] to draw any informative conclusions. In this scenario, it becomes clear that a thorough genomic characterization focused on a large cohort of highly proliferating carcinoid tumours in strict comparison with LCNECs and SCLCs is eagerly awaited.

In conclusion, although the histological subtyping still represents the cornerstone in lung carcinoid classification, the inter- and intra-tumour heterogeneity of proliferation indices together with different clinical behaviour within the same histological subgroups suggest that the current morphology-based system could be suboptimal for an effective prognostic stratification and therapy planning. Herein, we indicate that a subgroup of ACs showed brisk Ki-67 indices within the spectrum of allowed mitotic count. These patients were associated with specific clinical and pathological characteristics, to some extent regardless of histological subtyping. Preliminary data obtained by our group in thymus ACs on synchronous and metachronous lesions suggest that a progression capability of these lesions may stem form a markedly heterogeneous intra-tumour distribution of Ki-67 labelling indices. The correct identification of these tumours is clinically warranted. A thorough molecular characterization of this specific high proliferative index carcinoid subgroup may uncover the determinants of their biology and/or progression capacity, as well as identify biomarkers of potential therapeutic usefulness.
